# Associations between long-term ozone exposure and small airways function in Chinese young adults: a longitudinal cohort study

**DOI:** 10.1186/s12931-024-02679-4

**Published:** 2024-02-28

**Authors:** Shurong Feng, Liu Yang, Siqi Dou, Xinyuan Li, Shuo Wen, Lailai Yan, Wenzhong Huang, Yiwen Zhang, Bin Ma, Linghong Yuan, Shanshan Li, Peng Lu, Yuming Guo

**Affiliations:** 1 School of Public Health, Binzhou Medical University, 346 Guanhai Road, Yantai, 264003 Shandong China; 2 Department of Laboratorial Science and Technology, School of Public Health, Peking University, Beijing, China; 3 Climate, Air Quality Research Unit, School of Public Health and Preventive Medicine, Monash University, Level 2, 553 St Kilda Road, Melbourne, VIC 3004 Australia

**Keywords:** Lung function, Ozone, Cohort study, Long-term exposure

## Abstract

**Background:**

Increasing evidence is appearing that ozone has adverse effects on health. However, the association between long-term ozone exposure and lung function is still inconclusive.

**Objectives:**

To investigate the associations between long-term exposure to ozone and lung function in Chinese young adults.

**Methods:**

We conducted a prospective cohort study among 1594 college students with a mean age of 19.2 years at baseline in Shandong, China from September 2020 to September 2021. Lung function indicators were measured in September 2020 and September 2021, including forced vital capacity (FVC), forced expiratory volume in 1 s (FEV1), forced expiratory flow at the 25th, 50th, and 75th percentile of the FVC (FEF25, FEF50, and FEF75) and mean flow rate between 25% and 75% of the FVC (FEF25-75) were measured. Daily 10 km×10 km ozone concentrations come from a well-validated data-fusion approach. The time-weighted average concentrations in 12 months before the lung function test were defined as the long-term ozone exposure. The associations between long-term ozone exposure and lung function indicators in Chinese young adults were investigated using a linear mixed effects model, followed by stratified analyses regarding sex, BMI and history of respiratory diseases.

**Results:**

Each interquartile range (IQR) (8.9 µg/m^3^) increase in long-term ozone exposure were associated with a -204.3 (95% confidence interval (CI): -361.6, -47.0) ml/s, -146.3 (95% CI: -264.1, -28.4) ml/s, and − 132.8 (95% CI: -239.2, -26.4) ml/s change in FEF25, FEF50, and FEF25-75, respectively. Stronger adverse associations were found in female participants or those with BMI ≥ 24 kg/m^2^ and history of respiratory diseases.

**Conclusion:**

Long-term exposure to ambient ozone is associated with impaired small airway indicators in Chinese young adults. Females, participants with BMI ≥ 24 kg/m^2^ and a history of respiratory disease have stronger associations.

**Supplementary Information:**

The online version contains supplementary material available at 10.1186/s12931-024-02679-4.

## Introduction

China’s air quality has improved since national initiatives to combat air pollution were introduced in 2013 [1]. Between 2013 and 2020, there was a national decline in sulfur dioxide (SO_2_), nitrogen oxide (NO_X_) and primary fine particulate matter (PM_2.5_) emissions of roughly 69%, 28% and 44%, respectively [2]. However, China has experienced a continuous increase in annual ozone concentrations since 2013. The annual average ozone concentration has consistently exceeded 100 µg/m^3^, which is significantly higher than the recommended air quality standards set by various national and international organizations [3]. Possibly influenced by COVID-19, nitrogen oxide emissions have decreased [4]. The annual average ozone levels in 2020 and 2021 were 138 µg/m^3^ and 137 µg/m^3^ respectively in China (https://www.cma.gov.cn/zfxxgk/gknr/qxbg/). The level was 172 µg/m^3^ and 166 µg/m^3^ respectively in Shandong province (http://xxgk.sdein.gov.cn/xxgkml/hjzkgb/). There was a decreasing trend of O_3_ levels from 2020 to 2021.

Previous study suggests that low lung function in early adulthood is associated with incidence of later respiratory diseases [5]. Experimental evidence suggests that ozone exposure is associated with lung function decline. In a rat model, ozone exposure for six weeks led to the development of a chronic inflammatory process characterized by increased protease expression, epithelial cell apoptosis, and alveolar enlargement and destruction [6, 7]. A number of epidemiological studies have shown that short-term or long-term ozone exposure is associated with decreased lung function [7–10]. A longitudinal study of 3636 participants in the Multi-Ethnic Study of Atherosclerosis (MESA) showed that a 6.42 µg/m^3^ increase in long-term ozone exposure [mean ozone concentrations at baseline and follow-up years, median (range) of 10 (1–18) years] was significantly associated with decreases in FVC and FEV_1_ of 40.19 (95% CI: -17.88, -62.49) ml and 18.15 (95% CI: -1.59, -34.71) ml, respectively [8]. Another cross-sectional study of 50,991 adults aged over 20 in China has found that for every 1 SD increase in ozone concentration in the warm season, FEV 1 /FVC decrease by 0.3% (95% CI, 0.2–0.4%), FEF 50 decreases by 37.4 ml/s (95% CI, 24.8–50.0 ml/s), and FEF 75 decreases by 14.2 ml/s. s (95% CI, 8.8–19.6 ml/s) [11]. Another cross-sectional study of 40 nonsmoking elderly volunteers in China has found that every 10 µg/m^3^ increase in O_3_ levels, FEV1 and FVC decrease by 0.11 L (95% CI 0.02, 0.20) and 0.13 L (95% CI 0.01, 0.26) respectively [12]. Recently, a nationwide longitudinal study, conducted among 13,000 middle-aged and elderly people in China from 2011 to 2015, found that long-term exposure to warm season (April-September) O3 may reduce lung function [13]. A prospective cohort study of 3,014 participants drawn from 17 centers across eight countries found that a 7 µg/m³ (interquartile range) increase in the annual average of daily maximum 8-h average ozone concentrations was associated with a decline of FEV1 by − 2.08 mL/year (95% confidence interval [CI]: −2.79, − 1.36) and FVC by − 2.86 mL/year (95% CI, − 3.73, − 1.99) [14]. Few studies use cohort design to investigate the causal association between long-term ozone exposure and lung function in adults.

Therefore, based on the Chinese Undergraduates Cohort (CUC), we aim to evaluate the association between long-term ozone exposure and lung function among young adults, and to identify potentially vulnerable subpopulations.

## Methods

### Study design and study population

**Fig. 1:**
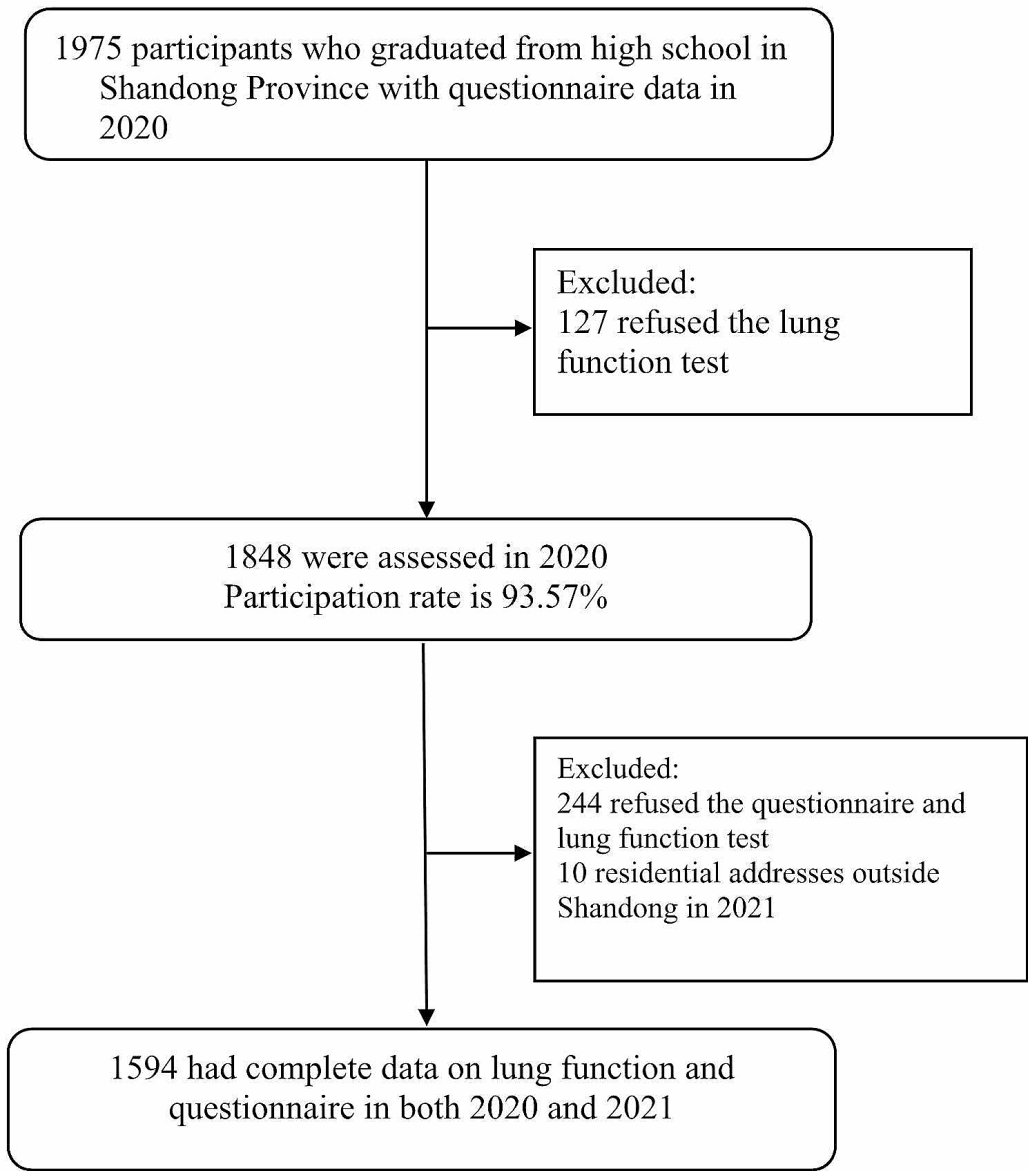
Flow chart of cohort study participants

The current study is based on the data from Chinese Undergraduates Cohort (CUC). The description of Chinese Undergraduates Cohort was detailed in previous studies [15, 16]. Briefly, Chinese Undergraduates Cohort is an ambispective cohort study designed to explore the health impacts of lifetime environmental exposures (e.g., air pollution, greenness, ambient temperature, and heavy metal markers) and behavioral factors. Participants with complete residential address recorded were recruited from Binzhou Medical University in Yantai, Shandong Province. The inclusion criteria are: (1) Enrolled in Binzhou Medical University in 2019; (2) High school addresses were in Shandong Province; (3) Residential addresses were within Shandong Province both 2020 and 2021; (4) Complete information on spirometry and questionnaires were available in both 2020 and 2021. The Exclusion criteria are: (1) Hearing or speech disabilities; (2) Serious oral or respiratory disease during lung function test. A final of 1,594 participants were included in this study (Fig. 1). The geographical distribution of the residential address of the study subjects was shown in Fig. S1. The study was approved by the Binzhou Medical University ethics committee and written informed consent were obtained from all study participants.

### Covariates

Standardized questionnaire on socio-demographic characteristics (e.g., age, sex, residential address, socioeconomic status), behavior and lifestyle (e.g., alcohol consumption, smoking status, passive smoking, physical activity), lung disease history were collected for each participant from September 29 to October 22, 2020 and September 1 to September 18, 2021, respectively. Smoking was defined as “at least one cigarette per day on average for more than six months” and passive smoking was defined as “ever having > 1 regular smoker in around, and being exposed > 15 minutes for at least 1 day per week” [17]. Alcohol consumption was defined as “at least one drink of alcohol per month on average in the past year” [18]. Physical activities were defined as “exercise at least once a week” [19]. The lung diseases history included the diagnosis of the following diseases: allergic rhinitis, chronic bronchitis, emphysema, pneumonia, tuberculosis and other lung diseases. Asthma was defined as the positive answer of “Have you ever had asthma diagnosed?” The definitions of other lung diseases were similar to asthma. The socioeconomic status was categorized into advantage and disadvantage groups according to the annual family income cutoff of 50,000RMB (≈ US$6894.17) [20].

### Lung function test

Details of the lung function tests were provided in our previous study [15, 16]. Lung function was measured in September 2020 and September 2021. Lung function tests were performed by well-trained professionals in accordance with the European Respiratory Society regulations [22]. Spirometers were calibrated before daily lung function test, no mouthpiece blockage, air leaks, premature termination or cut-off of exhalation during lung function test. Participants were instructed to perform between three and eight forced expiratory maneuvers until three acceptable flow-volume curves appeared to produce lung function results, keeping the best measurement results for analysis. Final include six lung function indicators: FVC, FEV1, forced expiratory flow at the 25th, 50th, and 75th percentile of FVC (FEF25, FEF50, and FEF75, respectively), and the mean flow rate between 25% and 75% of FVC (FEF25-75).

### Exposure assessment

Daily maximum 8-hour ambient ozone data between 2019 and 2021 with a spatial resolution of 10 km×10 km were obtained from Tracking Air Pollution in China (http://tapdata.org.cn) which is derived from machine learning models based on a data-fusion algorithm with predictors of ground measurements, satellite retrievals, emissions, chemical transport model simulations, and other sources [23]. It has a high quality (five-fold cross-validation coefficients of determination 0.70; root-mean-square error 26 µg/m^3^) and has been widely used in previous studies on health impact assessment of air pollution [24]. In order to adjust for potential confounding effects, we collected meteorological data (average temperature and relative humidity) of Shandong Province from the China Meteorological Data Sharing Service system (http://data.cma.gov.cn). The data were all station data, which mainly consisted of average temperature and relative humidity. We obtained the city-level daily average PM_2.5_ concentrations in Shandong Province from China National Environmental Monitoring Center (http://www.cnemc.cn/). The city level daily average PM_2.5_ concentrations was calculated based on the monitoring stations of the 16 cities in Shandong Province.

### Long-term ozone exposure

We defined the average of daily ozone exposure during 12 months before the lung function test as long-term exposure. Based on geocoded participants’ residential and school address, the overall ozone exposure for each individual was calculated as a time weighted average estimate combining the ozone data at residence and school [25, 26]:

$${\rm{E = }}\frac{{{C_r}*{T_r} + {C_s}*{T_s}}}{{{T_r} + {T_s}}}$$

where E is overall ozone exposure concentration, Cr and Cs are concentration at residence and school, respectively. T is approximate time spent at residence and school. For the participants, there are approximately 227 days (365 days a year, or 62.2%) at residence due to COVID-19 lockdown for 2020 and 112 days (30.7%) at residence for 2021. The variation of exposure is mainly driven by home address.

### Statistical methods

Given that each individual has multiple lung function tests during follow-up period, the association between ozone and lung function was analyzed using linear mixed-effects model. Potential confounders were selected based on previous relevant cohort studies [27, 28]. We developed two models: model 1: adjusted for sex, age, body mass index (BMI), lifestyle factors (smoking status, passive smoking, alcohol consumption, and physical activity), residential address (Urban vs. Rural), socioeconomic status, lung diseases history, temperature, and relative humidity. Temperature and relative humidity were introduced into the model with a natural cubic splines of 3 degree of freedoms to control their nonlinear effects [29]. Model 2: to control the effect of short-term ozone exposure on lung function, we further adjusted for the average ozone levels of the current day and the day before lung function test. Effect estimates were expressed as ml changes in FVC or FEV1, ml/s changes in FEF25, FEF50 FEF75 and FEF25-75 associated with an interquartile range (IQR) increase in ozone. The linear mixed effects model was constructed as follows.

$${\rm{Yit = \beta 1Xitj + \beta 2Timeit + }}\left( {{\rm{1|Participant}}} \right)$$

Yit represents the pulmonary function measurement result of survey subject i at the t-th follow-up visit. Xitj represents the covariate j of survey subject i at time t. Timeit is the measurement time of Yit. This study is Year 2020 and 2021. β2 represents the regression of Time Coefficients, where β1 and β2 are fixed effects. (1|Participant) is the random intercept of the participants, that is, each individual has a different intercept value.

Numerous previous toxicological studies have demonstrated the potential moderating effects of sex and BMI on ozone [30, 31]. First, we tested the interaction between BMI and sex and found no interactions (*p* > 0.05), so we conducted subgroup analyses stratified by sex and BMI at baseline (< 24 and ≥ 24 kg/m^2^). In addition, we further conducted a stratified analysis based on the history of respiratory diseases. We performed the following sensitivity analyses to assess the robustness of the results: There would be a combined effect if exposed to both smoking and air pollution [32]. To minimize the confounding effect of smoking, we excluded smoking participants. Based on Models 1, we included outdoor time to further test the robustness of the model. We included daily average PM_2.5_ concentration as covariates in Model 2 to check the robustness of the results.

We used generalized additive mixed models (GAMM) smooth functions and random effects to explore the exposure-response (E-R) relationship. In these models, a natural cubic spline was added for the long-term ozone concentration to allow for a possible nonlinear relationship [33, 34]. All analyses were performed in R software with the “nlme” package (version 4.0.5). The threshold for statistical significance was *p*-value < 0.05 for the 2-sided test.

## Results

### Subject’s characteristics

The general demographic characteristics and lung function test results of the study population in 2020 and 2021 are shown in Table 1. The participants had slight majority of females (56.1%)and few smokers (1.3%). There were 70 (4.4%) alcohol consumers. The mean age of study population was 19.2 years (SD = 0.7) and the mean BMI was 23.9 kg/m^2^ (SD = 3.8). There were 142 (8.9%) participants with self-reported lung disease history. The annual average ozone concentrations were 110.7 µg/m^3^ in 2020, and decreased to 102.0 µg/m^3^ in 2021.

**Table 1:** Demographic characteristics and lung function outcomes among participants (*N* = 1594)

	Year	
Participant characteristics	2020	2021
**Demographic characteristic**		
Age (years)	19.2 ± 0.7	20.2 ± 0.7
Female	894(56.1)	894(56.1)
Urban	841(52.8)	879(55.1)
BMI (kg/m^2^)	21.9 ± 3.8	21.3 ± 3.7
**Cigarette smoke exposure**		
Smoking status	20(1.3)	26(1.6)
Passive smoking	107(6.7)	130(8.2)
**Alcohol consumption (Yes)**	70(4.4)	81(5.1)
**Physical activity at least once a week (Yes)**	1285(80.6)	982(61.6)
**Lung disease history (Yes)**	142(8.9)	160(10.0)
**Socioeconomic status**		
Socioeconomic-disadvantage	581(36.4)	533(33.4)
Socioeconomic-advantage	1013(63.6)	1061(66.6)
**Lung function index**		
FVC (ml)	3158.8 ± 815.4	3420.5 ± 770.6
FEV1.0 (ml)	3005.3 ± 729.9	3182.9 ± 667.7
FEF25 (ml/s)	5716.7 ± 1846.1	5924.9 ± 1759.9
FEF50 (ml/s)	4685.9 ± 1370.6	4581.2 ± 1312.5
FEF75 (ml/s)	3043.9 ± 1010.3	2745.1 ± 937.6
FEF25-75 (ml/s)	4374.5 ± 1256.1	4219.6 ± 1177.8
**Ozone (µg/m** ^**3**^ **)**	110.7	102.0

Note: results are expressed as n (%)

Table 2 shows the lung function among different subgroups. Males (*n* = 700) and BMI ≥ 24 kg/m^2^ (*n* = 371) groups had higher lung function than females (*n* = 894) and BMI < 24 kg/m^2^ (*n* = 1223) in 2020 and 2021. During follow-up, FVC, FEV1 and FEF25 increased in all subgroups.

**Table 2:** Description of lung function stratified by sex and BMI in 2020 and 2021

Variables	FVC (ml)	FEV1 (ml)	FEF25 (ml/s)	FEF50 (ml/s)	FEF75 (ml/s)	FEF25-75 (ml/s)
**During 2020**						
**Sex**						
Male	3708.8 ± 744.4	3514.9 ± 642.4	6836.3 ± 1762.2	5442.3 ± 1361.3	3436.2 ± 1093.4	5066.5 ± 1246.7
Female	2728.0 ± 573.9	2606.2 ± 514.2	4840.1 ± 1377.2	4093.7 ± 1049.2	2736.6 ± 818.4	3832.6 ± 963.6
**BMI**						
< 24 kg/m^2^	3052.2 ± 767.7	2913.8 ± 694.8	5512.7 ± 1772.5	4544.0 ± 1325.1	3007.9 ± 995.6	4258.7 ± 1221.9
≥ 24 kg/m^2^	3510.3 ± 868.8	3307.0 ± 761.8	6389.2 ± 1924.9	5153.9 ± 1415.1	3162.5 ± 1049.9	4756.0 ± 1292.8
**During 2021**						
**Sex**						
Male	4068.7 ± 597.7	3761.6 ± 495.2	7154.5 ± 1629.2	5354.8 ± 1347.0	3102.4 ± 1044.7	4900.8 ± 1212.1
Female	2913.0 ± 438.7	2729.9 ± 368.0	4962.1 ± 1155.3	3975.4 ± 903.5	2465.4 ± 731.9	3686.2 ± 822.1
**BMI**						
< 24 kg/m^2^	3296.7 ± 727.1	3085.7 ± 641.1	5715.1 ± 1686.9	4449.5 ± 1250.4	2734.5 ± 921.9	4123.2 ± 1135.8
≥ 24 kg/m^2^	3828.4 ± 769.9	3503.4 ± 653.8	6616.7 ± 1819.8	5354.8 ± 1416.7	2780.2 ± 988.2	4537.7 ± 1256.8

Note: Results are expressed as Mean ± SD; SD, standard deviation

### Associations between long-term exposure to ozone and lung function

**Fig. 2:**
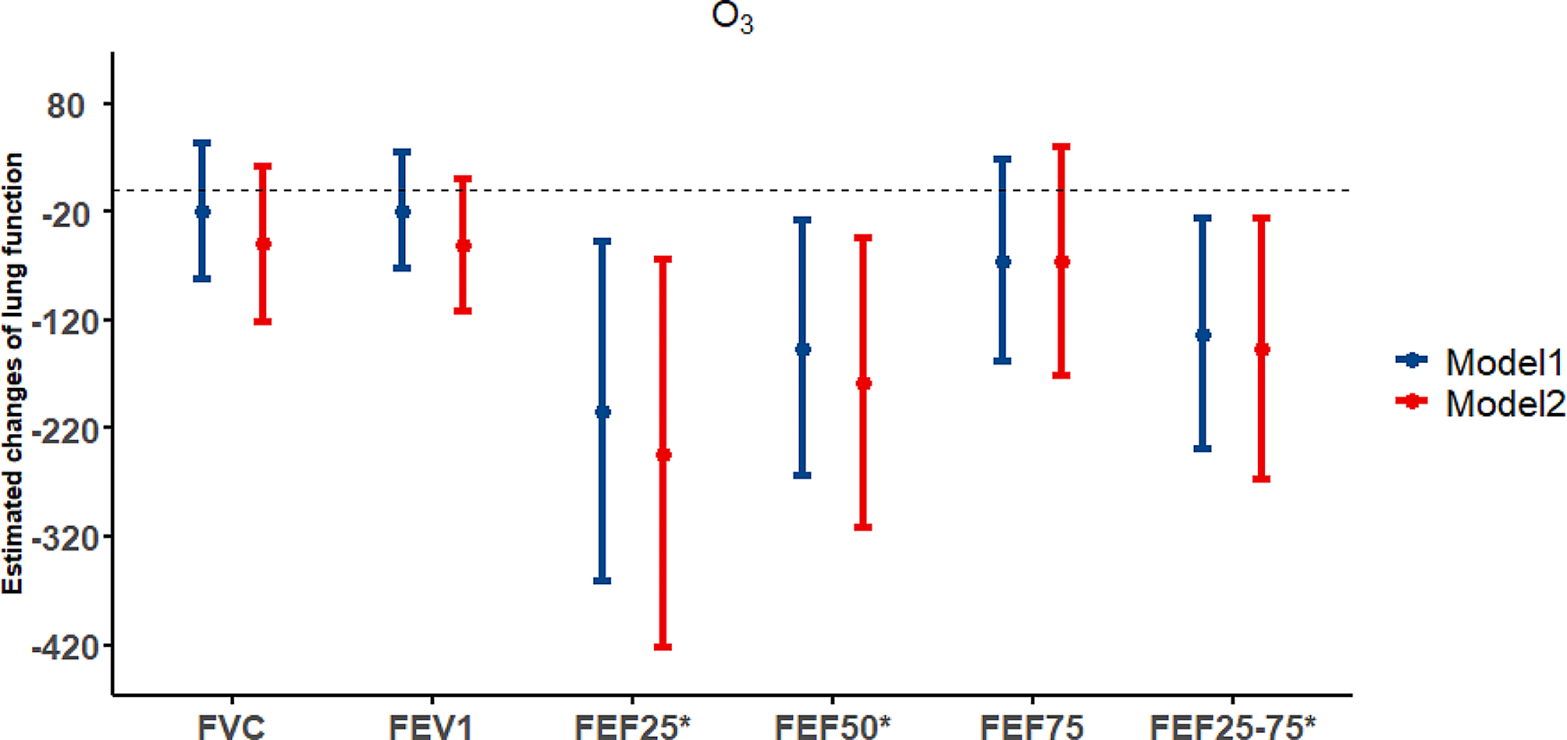
The association between IQR increase in the ozone annual pollutant concentrations and changes in lung function parameters. Note: * represents *P* < 0.05. The Model1 adjusted for ozone, sex, age, BMI, lifestyle factors (smoking status, alcohol drinking, physical activity), respiratory disease history, residential address (rural or urban areas), socioeconomic status, temperature, relative humidity; The Model2 adjusted for short-term ozone exposure (lag01) based on the above

Figure 2 shows the associations between long-term ozone exposure and lung function. We consistently observed that annual ozone concentrations were significantly associated with decreased FEF25, FEF50 and FEF25-75. Each IQR increase in annual ozone concentrations was associated with decrements of 204.3 (95% CI: -361.6, -47.0) ml/s in FEF25, 146.3 (95% CI: -264.1, -28.4) ml/s in FEF50, and 132.8 (95% CI: -239.2, -26.4) in FEF25-75. We did not observe any significant associations with FVC, FEV1 and FEF75. After controlling for short-term ozone exposure, the associations with FEF25, FEF50 and FEF25-75 remained statistically significant. In addition, we observed that the E-R curves of ozone and lung function are nearly linear (Figure. S2).

### Stratified analyses

**Fig. 3:**
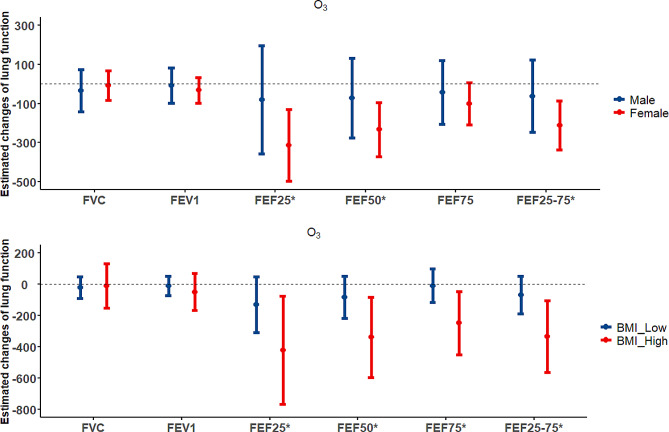
The association between IQR increase in the ozone annual pollutant concentrations and changes in lung function parameters among participants stratified by sex (males and females) and BMI (< 24 and ≥ 24 kg/m^2^). Note: * represents *P* < 0.05

Figure 3 shows the results of the stratification analyses by gender and BMI. We found that the association between ozone and lung function was stronger in women than in men. For example, with every IQR increase in annual ozone concentrations, FEF25 decreased by 311.1 ml/s (95% CI: -492.6, -129.6 ml/s), FEF50 by 232.2 ml/s (95% CI: -370.0, -94.4 ml/s), FEF25-75 decreased by 209.4 ml/s (95% CI: -333.4, -85.5 ml/s) in females, while the corresponding changes were statistically insignificant for males. In addition, we observed a stronger association in participants with BMI ≥ 24 kg/m^2^. Each IQR increase in annual ozone concentrations was associated with 421.0 ml/s (95% CI: -764.1,-77.9 ml/s) decline of FEF25, 337.3 ml/s (95% CI: -591.5,-83.0 ml/s) decline of FEF50 and 332.3 ml/s (95% CI: -560.8,-103.9 ml/s) decline of FEF25-75, and such associations were not observed in BMI<24 kg/m^2^ participants. Fig. S3 shows the results of the stratification analyses by history of respiratory diseases. We found that the association between ozone and small airway function was stronger in participants with a history of respiratory disease. For example, with every IQR increase in annual ozone concentrations, FEF25 decreased by 521.9 ml/s (95% CI: 913.3,-130.5 ml/s), FEF50 by 493.4 ml/s (95% CI: -798.9,-187.9 ml/s), FEF75 by 322.1 ml/s (95% CI: -563.8,-80.3 ml/s), and FEF25-75 by 410.5 ml/s (95% CI: -687.4,-133.5 ml/s) in participants with a history of respiratory disease, while the corresponding changes were statistically insignificant for participants without respiratory disease.

### Sensitivity analyses

After excluding smoking participants from the model, the results remained robust. The estimated decrease of FEF25 with an IQR increase of ozone concentration was 204.3 ml/s (95% CI: -361.6, -47.0 ml/s), compared with 210.2 ml/s (95% CI: -368.2, -52.3 ml/s) after excluding smoking participants (Table S6). After including outdoor time and PM_2.5_ into the model, the results remain robust (Figure S4).

## Discussion

As far as we know, this is the first cohort study to evaluate the association between long-term ozone exposure and lung function in young adults in China. Our results identified consistent associations between long-term ozone exposure and decreased small airway functions such as FEF25, FEF50 and FEF25-75. In the subgroup analysis, we found that these associations were more prominent in female, BMI ≥ 24 kg/m^2^ population and those with respiratory diseases histories.

Few previous studies have estimated the impact of the exposure to ozone on small airways. Small airways are airways with a lumen diameter less than 2 mm during inspiration [35]. The small airways can become compromised before the proximal airways become obstructed and/or before any symptoms appear. It is considered to be a precursor to common respiratory diseases, such as chronic obstructive pulmonary disease (COPD) and asthma [36, 37]. Studies have shown that FEF25, FEF50, and FEF25-75 are feasible indicators for early identification of small airway function [38]. Studies have found that ozone has adverse effects on lung function, and it is particularly important to investigate the effects of ozone on small airways of young people and to understand how ozone exposure affects the respiratory system at an early stage [39, 40]. In our study, we found that exposure to ambient ozone had statistically negative significant associations with three indicators of small airways function (FEF25, FEF50, FEF25-75). The results of previous studies were consistent with our study. A cross-sectional study of German adolescents found negative effects of ozone exposure on FEF25, FEF50 and FEF25-75 [41]. A recent cross-sectional study by Niu et al. using 50,991 participants from the China Pulmonary Health (CPH) study to explore whether ozone exposure impairs lung function found an independent association between long-term ozone exposure and impaired small airway function. Each 1 SD (10.5 µg/m^3^) increase in ozone was associated with a 37.4 (95% CI: 24.8, 50.0) ml/s and 29.5 (95% CI: 19.6, 39.5) ml/s decrease in FEF50 and FEF25-75, respectively [11]. Our estimated effects of ozone exposure on FEF50 and FEF25-75 [ -146.3 (95% CI: -264.1, -28.4) ml/s and − 132.8 (95% CI: -239.2, -26.4) ml/s per IQR (8.9 µg/m^3^) increase in ozone] were stronger than the results of Niu et al. This discrepancy may be attributable to the higher ozone concentrations reported in our study (mean: 110.7 µg/m^3^ vs. 90.1 µg/m^3^). Studies have shown that the lung function parameters of participants exposed to high concentrations decreased more rapidly than those exposed to low concentrations of ozone [42]. In addition, the ozone exposure time window of Niu et al. was average concentration of ozone from May to October, while our ozone concentration was the annual average concentration. Different exposure time may lead to different results. Difference in study populations may also partly explain the differences in results, which may vary by individual’s physical condition, lifestyle, education, and activity pattern. It is difficult to directly compare our effect size with other studies, given the differences in study design, target participants and statistical methods. Nevertheless, our study provides new evidence that long-term exposure to ozone can impair small airways.

This study found no association between ozone exposure and FEF75. FEF 75% refers to the flow when 75% of FVC has been exhaled. Studies have pointed out that different vital capacity indicators reflect the airflow characteristics of different airways. FEF25 reflects the airflow characteristics of small airways, while FEF75 corresponds to large airways [2]. Pathological evidence from animal studies suggests that the central acinar region and/or terminal bronchioles are more susceptible to ozone toxicity [3]. Therefore, ozone is more likely to have adverse effects on small airways, causing FEF25 to decrease. Due to its low water solubility, inhaled ozone will remain in the respiratory system and damage the health of small airways in the long term [4]. Possibly for this reason, we did not find positive associations with FEF75 in this study.

Moreover, our study did not find a statistically significant association between ozone exposure and FEV1 and FVC. In consistent with our findings, Yue Niu et al. [11] also did not find statistically significant associations between long-term ozone exposure and FEV1 and FVC. Additionally, pathological evidence from animal studies suggests that the central acinar region and/or terminal bronchioles are more susceptible to ozone toxicity compared to the proximal airways [43–45]. Furthermore, our study had a relatively short duration, which may have precluded the observation of the relationship between ozone exposure and proximal airways. We will continue to investigate the relationship between long-term ozone exposure and lung function in future studies.

The mechanism by which ozone damages the small airways remains unclear. But animal studies revealed that ozone was more likely to damage central and terminal bronchi, and its effects on small airways last longer [46]. Additionally, ozone was inadequately cleared by the upper respiratory tract due to its low water solubility, it may remain in the respiratory system [47]. As a result, the lower respiratory tract accumulates the most of the inhaled ozone [47]. These may lead to long-term ozone hazards to the lower respiratory tract.

In the sex stratified analysis, we found female’s lung function appeared to be more susceptible to ozone than male. Females are reported to be more susceptible to inflammatory lung diseases caused by air pollution than males. A repeated measure study of children in Tianjin, China, showed an obvious sex difference, suggesting that ozone has a more prominent effect on the female group [48]. This is consistent with our results. Toxicological evidence suggested there are gender differences in lung impairment caused by ozone [49]. Fuentes et al. measured the mice for changes in lung function and inflammatory gene expression after gonadectomy in female and male mice exposed to ozone. They found that in female mice, gonadectomy reduced ozone-induced airway hyperresponsiveness (AHR) and lung interleukin 6 (IL-6) expression. Suggesting that female gonadal hormone levels could possibly mediate an inflammatory response in the lungs. However, the gonadectomy male mice showed higher expression of AHR and inflammatory genes compared to controls [50]. This may be one of the reasons for the sex differences in ozone induced lung inflammation and injury. This finding suggests that we should pay attention to the health effects of ozone, especially in females.

In the BMI stratified analysis, we found the association between ozone and lung function impairment was stronger for participants with BMI ≥ 24 kg/m^2^. In consistent with our results, a cross-sectional study by Doiron et al. in the United Kindom found that being overweight or obese deteriorated the effects of air pollution on adult lung function [51]. The Seven Northeastern Chinese Cities (SNEC) study also found that obese individuals were more susceptible to the adverse effects of air pollutants on lung function [52]. Obesity may lead to significant changes in lung and chest wall mechanics with age due to excessive fat deposits in the diaphragm, chest wall and abdominal cavity [53, 54]. These mechanical changes can lead to restrictive ventilatory dysfunction by reducing lung and chest wall compliance [53]. Toxicological evidence found that ozone causes an increase in pulmonary resistance (RL) in obese mice, but not in lean mice [55]. In addition, ozone caused a greater increase in bronchoalveolar lavage neutrophils and AHR in obese mice compared with lean mice [55]. Our study further confirmed that the lung function of overweight and obese people was more vulnerable to ozone exposure. Overweight people, especially obese people, should pay more attention to self-protection. In the stratified analysis for history of respiratory disease, we found that the association between ozone and small airway function impairment was stronger in participants with a history of respiratory disease. Similarly, previous studies reported that participants with respiratory diseases may be more susceptible to ozone exposure [11, 56, 57]. Further investigations are needed to examine the mechanistic explanations.

This study had many strengths. The nature of the cohort allowed us to longitudinally investigate the relationship of ozone exposure on lung function. Furthermore, the 10 km×10 km grid data was used to estimate ozone exposure, which allowed us to overcome the spatial coverage limitations that typically arose with monitoring stations. Thirdly, the stratification analysis allowed us to understand sensitive populations more accurately.

However, this study also has some limitations. Firstly, the study participants are young college students from Shandong, China. They cannot represent the Chinese population. The generalizability of our findings may be limited. Secondly, the exposure level of ozone was assigned to the participant’s fixed address. There was no information about the patterns of daily activities. Thirdly, self-reported questionnaires may have recall bias. Besides, the 10 km × 10 km grid data used in our study is broad, and we will consider using more accurate data in future studies. Finally, even though we designed the questionnaire as detailed as possible, we still missed some information such as data on parental factors and data on epidemic-related mental stress, we cannot rule out residual confounding by these and other factors.

## Conclusions

Long-term ozone exposure may impair small airway lung function indicators in young adults. Females, overweight participants and those with respiratory diseases history are more sensitive to ozone exposure. These results indicate that ozone could possibly affect small airways through the metabolic or sex-hormone relevant pathways.

### Electronic supplementary material

Below is the link to the electronic supplementary material.

Supplementary Material 1

## Data Availability

The datasets used and analyzed during the current study are available from the corresponding author on reasonable request.
