# Supplementary material for: Associations between long-term ozone exposure and small airways function in Chinese young adults: a longitudinal cohort study
Source: Respir Res. 2024 Feb 28;25:105. doi: 10.1186/s12931-024-02679-4 (PMC10902944; doi:10.1186/s12931-024-02679-4)
Supplement: Supplementary file 1 — Supplementary Material 1 [file 12931_2024_2679_MOESM1_ESM.docx]

**Associations between Long-term Ozone Exposure and Small Airways Function in Chinese Young Adults: A Longitudinal Cohort Study**

**Supplementary Material**

**
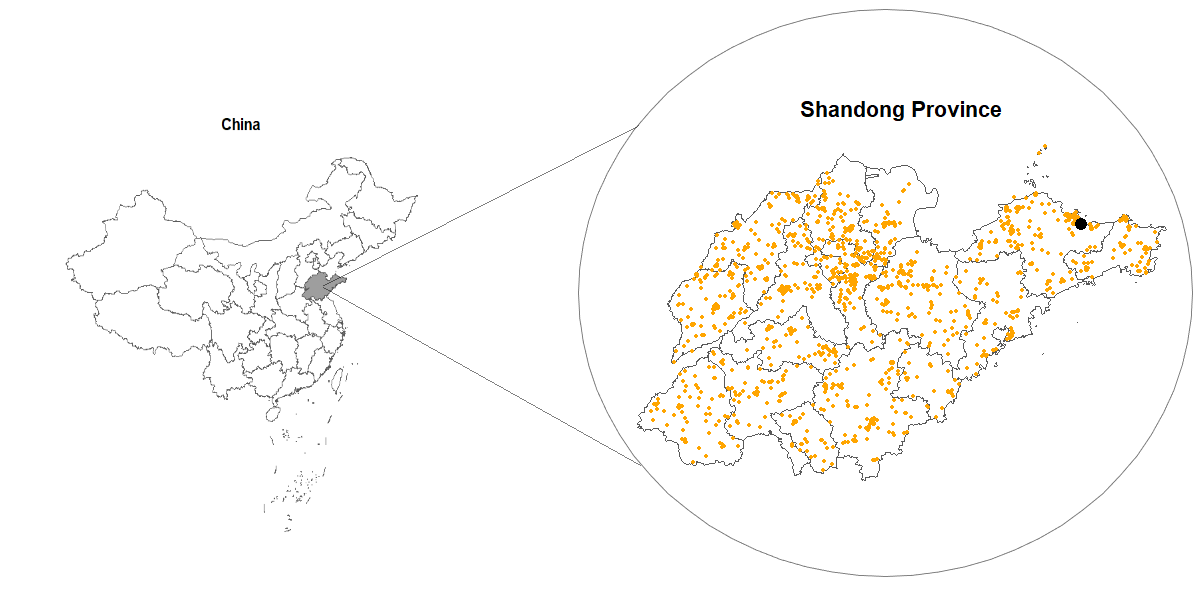
**

**Fig. S1** Geographical distribution of the study population

Note: The gray area on the left represents Shandong Province; The yellow dots on the right represent participants' residential address and the black dot represents the school address.


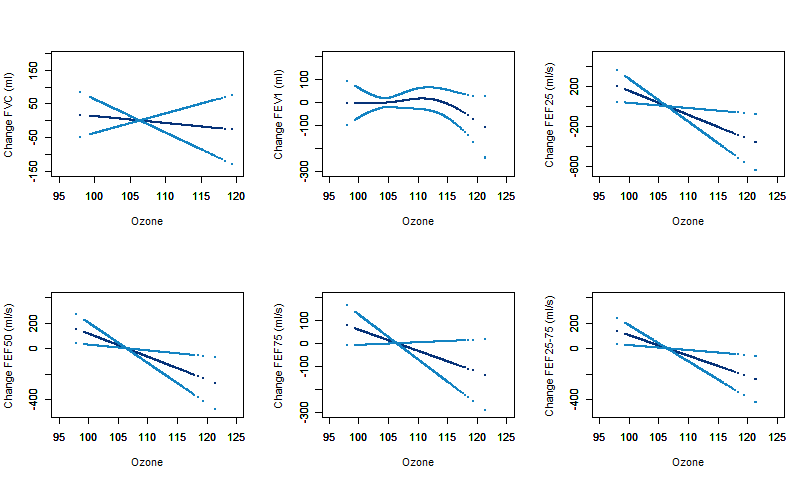
**Figure. S2**. Exposure–Response curves for the relationships of Ozone with lung function parameters. The Y-axes for lung function denote changes in these parameters.


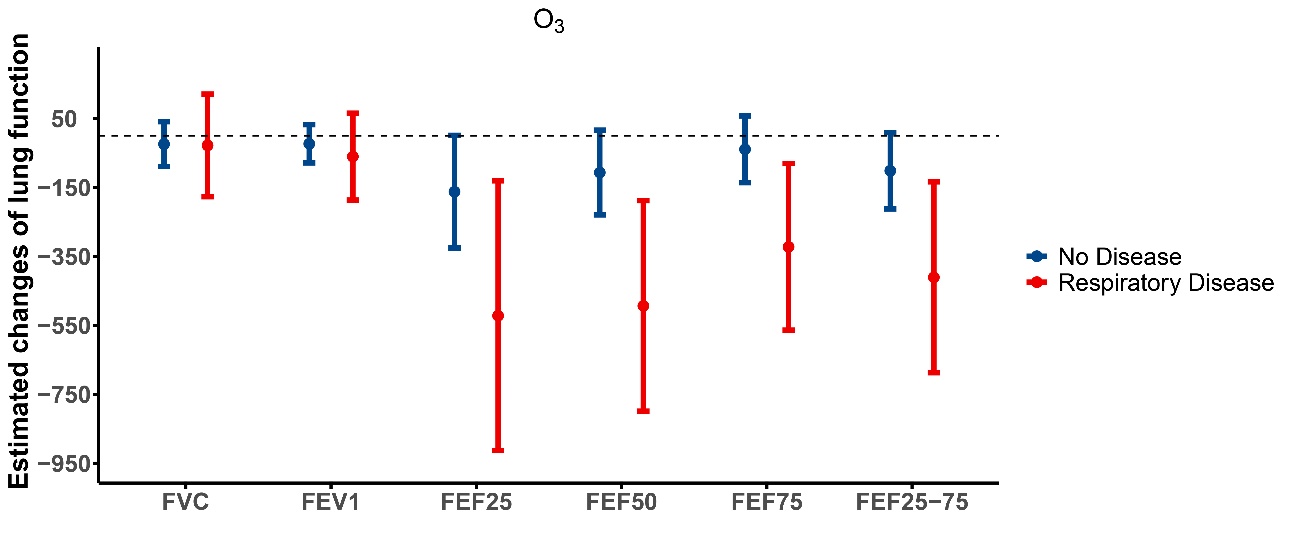


**Figure. S3**. The association between IQR increase in the ozone annual pollutant concentrations and changes in lung function parameters among participants stratified by respiratory diseases history.

**Table S1.** A comparison of the characteristics for included and non-included participants within the cohort.

| **characteristics** | **Included (1594)** | **non-included (254)** | ***P* for difference** |
| --- | --- | --- | --- |
| **Demographic characteristic** |  |  |  |
| Age (years) | 19.2 ± 0.7 | 19.2 ± 0.6 | 0.55 |
| Female (%) | 894 (56.1） | 130 (51.2） | 0.16 |
| Urban (%) | 841 (52.8） | 120 (47.2） | 0.12 |
| BMI (kg/m^2^) | 23.9 ± 3.8 | 22.1 ± 3.9 | 0.27 |
| **Cigarette smoke exposure (%)** |  |  |  |
| Smoking status | 20 (1.3） | 7 (3.9) | 0.08 |
| Passive smoking | 107 (6.7） | 12 (4.7) | 0.29 |
| **Alcohol consumption (Yes)** | 70 (4.4) | 17 (6.7) | 0.15 |
| **Physical activity at least once a week (%) (Yes)** | 1285 (80.6) | 193 (75.8) | 0.10 |
| **Lung disease history (%) (Yes)** | 142 (8.9) | 33 (13.0) | 0.05 |
| **Socioeconomic status (%)** |  |  |  |
| Socioeconomic-disadvantage | 581 (36.4） | 99 (39.0) | 0.48 |
| Socioeconomic-advantage | 1013 (63.6) | 155 (61.0) |  |

**Table S2.** Baseline and follow-up ozone concentrations.

| **Ozone (μg/m^3^)** | **Year** | | |
| --- | --- | --- | --- |
|  | 2020 |  | 2021 |
| **Minimum** | 87.4 |  | 98.8 |
| **Mean ± Sd** | 110.7 ± 4.2 |  | 102.0 ± 1.2 |
| **Median** | 111.1 |  | 102.3 |
| **Interquartile range** | (107.9,113.9) |  | (101.0,103.0) |
| **Maximum** | 121.4 |  | 15.15 |

**Table S3**. Associations between IQR increase in the average ozone concentrations and changes in lung function parameters in young adults.

| **Lung Function** | **Model1** | | **Model2** | |
| --- | --- | --- | --- | --- |
|  | Difference (95% CI) | *p* | Difference (95% CI) | *p* |
| **FVC** (ml) | -19.2 (-82.2,43.8) | 0.55 | -49.8 (-121.6,21.9) | 0.17 |
| **FEV1** (ml) | -19.2 (-73.1,34.7) | 0.49 | -50.6 (-111.9,10.8) | 0.11 |
| **FEF25** (ml/s) | -204.3 (-361.6,-47) | 0.01 | -243.6 (-422.7,-64.4) | 0.01 |
| **FEF50** (ml/s) | -146.3 (-264.1,-28.4) | 0.02 | -177.7 (-311.4,-43.9) | 0.01 |
| **FEF75** (ml/s) | -65.0(-158.1,28.0) | 0.17 | -66.2 (-171.9,39.5) | 0.22 |
| **FEF25-75** (ml/s) | -132.8 (-239.2,-26.4) | 0.01 | -146.9 (-267.6,-26.1) | 0.02 |

Note: The Model1 adjusted for ozone, sex, age, BMI, lifestyle factors (smoking status, alcohol drinking, physical activity), respiratory disease history, home location (rural or urban areas), socioeconomic status, temperature, relative humidity; The Model2 adjusted for short-term ozone exposure (lag01) based on the above.

**Table S4**. The association between IQR increase in the average ozone concentrations and changes in lung function parameters stratified by sex.

| **Lung Function** | **Male** | | **Female** | |
| --- | --- | --- | --- | --- |
|  | Difference (95% CI) | *p* | Difference (95% CI) | *p* |
| **FVC** (ml) | -34.4 (-140.8,72) | 0.53 | -7.8 (-82.3,66.7) | 0.84 |
| **FEV1** (ml) | -8.0 (-98.4,82.3) | 0.86 | -32.2 (-97.1,32.7) | 0.33 |
| **FEF25** (ml/s) | -79.9 (-355.1,195.4) | 0.57 | -311.1 (-492.6,-129.6) | <0.01 |
| **FEF50** (ml/s) | -72.1 (-274.6,130.4) | 0.49 | -232.2 (-370.0,-94.4) | <0.01 |
| **FEF75** (ml/s) | -42.7 (-205.3,119.9) | 0.61 | -101.1 (-208.3,6.2) | 0.07 |
| **FEF25-75** (ml/s) | -62.3 (-246.2,121.5) | 0.51 | -209.4 (-333.4,-85.5) | <0.01 |

**Table S5**. The association between IQR increase in the average ozone concentrations and changes in lung function parameters stratified by BMI.

| **Lung Function** | **BMI_Low** |  | **BMI_High** |  |
| --- | --- | --- | --- | --- |
|  | Difference (95% CI) | *p* | Difference (95% CI) | *p* |
| **FVC** (ml) | -22 (-91.9,48.0) | 0.54 | -11.0 (-152.4,130.5) | 0.88 |
| **FEV1** (ml) | -10.6 (-71.1,50.0) | 0.73 | -49.6 (-167.9,68.6) | 0.41 |
| **FEF25** (ml/s) | -129.2 (-306.3,48) | 0.15 | -421.0 (-764.1,-77.9) | 0.02 |
| **FEF50** (ml/s) | -84.0 (-217.2,49.2) | 0.22 | -337.3 (-591.5,-83) | 0.01 |
| **FEF75** (ml/s) | -9.8 (-115.1,95.5) | 0.86 | -246.5 (-447.1,-45.9) | 0.02 |
| **FEF25-75** (ml/s) | -68.7 (-189.3,51.8) | 0.26 | -332.3 (-560.8,-103.9) | <0.01 |

Note: BMI_Low: <24kg/m^2^; BMI_High: ≥24 kg/m^2^

**Table S6.** The association between IQR increase in the average ozone concentrations and changes in lung function parameters After excluding smoking participants.

| **Lung Function** | **Excluding smoker** | |
| --- | --- | --- |
|  | Difference (95% CI) | *p* |
| **FVC** (ml) | -11.3 (-74.5,51.8) | 0.73 |
| **FEV1** (ml) | -13.8 (-67.9,40.3) | 0.62 |
| **FEF25** (ml/s) | -210.2 (-368.2,-52.3) | 0.01 |
| **FEF50** (ml/s) | -149.3 (-267.6,-30.9) | 0.01 |
| **FEF75** (ml/s) | -63.7 (-157.3,30.0) | 0.18 |
| **FEF25-75** (ml/s) | -133.7 (-240.1,-27.4) | 0.01 |


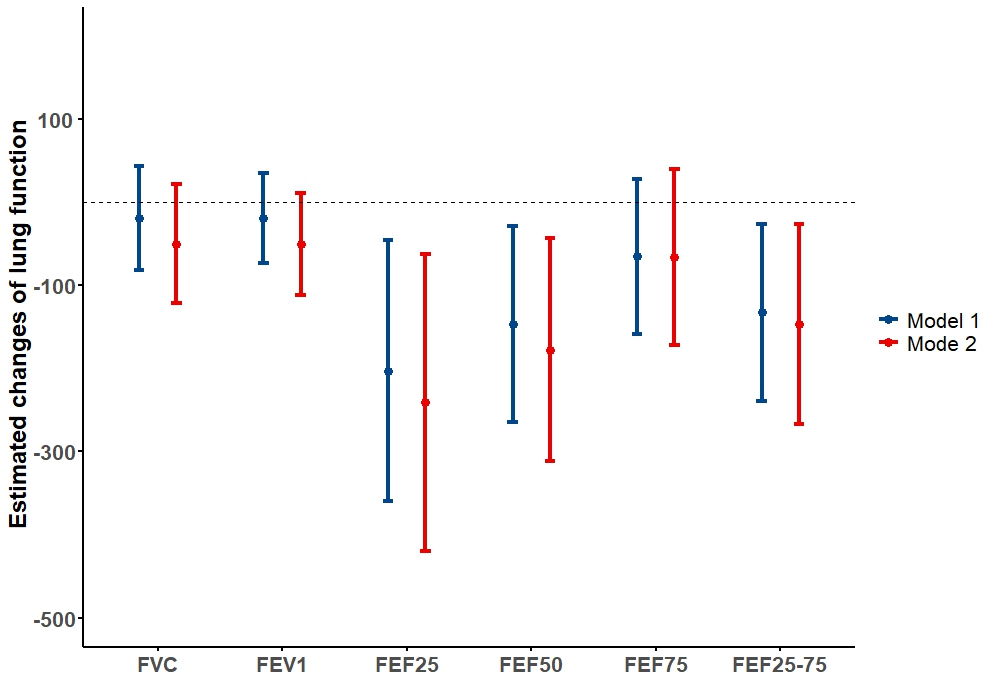


**Figure. S4** The association between IQR increase in the ozone annual pollutant concentrations and changes in lung function parameters.

Note: * represents *P* < 0.05. The Model 1 adjusted for ozone, sex, age, BMI, lifestyle factors (smoking status, alcohol drinking, physical activity, outdoor time), respiratory disease history, residential address (rural or urban areas), socioeconomic status, temperature, relative humidity; The Model 2 adjusted for short-term ozone exposure (lag01) based on the above.
